# Tetraspanins affect membrane structures and the trafficking of molecular partners: what impact on extracellular vesicles?

**DOI:** 10.1042/BST20240523

**Published:** 2025-03-26

**Authors:** Eric Rubinstein, Clotilde Théry, Pascale Zimmermann

**Affiliations:** 1 Centre d'Immunologie et des Maladies Infectieuses (CIMI-Paris), Sorbonne Université, Inserm, CNRS, Paris, France; 2 Institut Curie Research Center, PSL Research University, INSERM U932, Paris, France; 3 Institut Curie Research Center, CurieCoreTech Extracellular Vesicles, Paris, France; 4 Equipe labellisée Ligue 2024, Centre de Recherche en Cancérologie de Marseille (CRCM), Aix-Marseille Université, Inserm, CNRS, Institut Paoli-Calmettes, Marseille, France; 5 Department of Human Genetics, KU Leuven, Leuven, Belgium

**Keywords:** tetraspanins, microparticles, membrane dynamics, membrane proteins, exosomes, extracellular vesicles

## Abstract

Tetraspanins are a family of 33 proteins in mammals believed to play a crucial role in the compartmentalization of various associated proteins within cells and membranes. Recent studies have elucidated the structure of several tetraspanin members, revealing that while the four transmembrane domains typically adopt a cone-shaped configuration in crystals, other conformations are also possible. This cone-shaped structure may explain why tetraspanins are often enriched in curved and tubular cellular structures, such as microvilli, tunneling nanotubes, retraction fibers, or at the site of virus budding, and may contribute to the formation or maintenance of these structures. Tetraspanins have also been detected on midbody remnants and migrasomes, as well as on extracellular vesicles (EVs), for which CD9, CD81, and CD63 are widely used as markers. Although their impact on certain membrane structures and their ability to regulate the function and trafficking of associated proteins would suggest a potential role of tetraspanins either in EV formation or in regulating their protein composition, or both, efforts to characterize these roles have been complicated by conflicting results. In line with the interaction of certain tetraspanins with cholesterol, two recent studies have suggested that the presence or organization of oxysterols and cholesterol in EVs may be regulated by Tspan6 and CD63, respectively, paving the way for further research on the influence of tetraspanins on the lipid composition of EVs.

## Introduction

Tetraspanins are a family of proteins characterized by four transmembrane domains, expressed across all metazoans, and consist of 33 members in mammals [1,2]. Genetic studies in mice and the identification of rare mutations in humans have underscored the critical roles of specific tetraspanins in various biological processes, including immunity, vision, kidney function, fertilization, muscle regeneration, and the regulation of distinct cell populations within various organs (for review [3–5]). Additionally, numerous studies have highlighted their involvement in cellular infection by intracellular pathogens and the regulation of cancer progression (for review [6,7]). Here, we discuss the role of tetraspanins on membrane organelles and trafficking, with a focus on extracellular vesicles (EVs).

**Figure 1:**
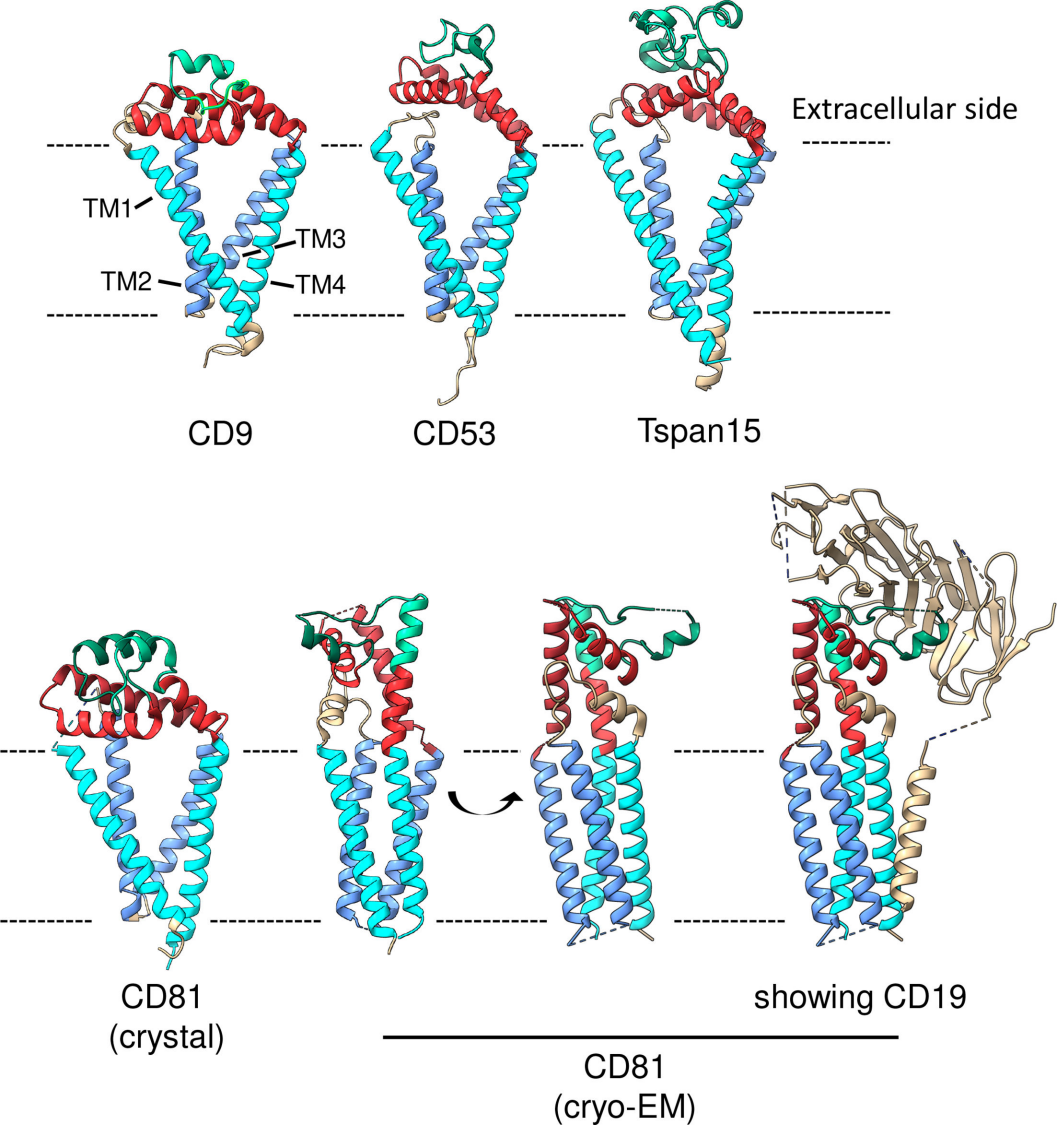
Structure of four tetraspanins. The four transmembrane domains are in blue, the three structurally conserved helices in the large extracellular domain in red, and the variable region in green. The small extracellular domain is in gold. All structures were downloaded from PDB (www.rcsb.org). Top: the crystal structures of CD9 (PDB # 6K4J) and CD53 (PDB # 6WVG) and the cryo-EM structure of Tspan15 in association with ADAM10 (not shown). Bottom: the structure of CD81 (PDB # 5TCX) in crystals and that determined by cryo-EM (PDB # 7JIC) in which CD81 is in complex with CD19. Three different views of this structure are shown, including one showing CD19. Note that in this structure, CD81 does not show an intramembrane cavity and that the LEL extends from the plane of the membrane.

Tetraspanins are relatively small proteins, with a backbone of 200–300 amino acids but are variably glycosylated. In addition to their four transmembrane domains, tetraspanins share several structural features, including conserved residues, particularly cysteines. Intracellular cysteines serve as sites for palmitoylation, while extracellular cysteines contribute to the specific folding of the larger one of the two extracellular domains [4,5,8]. Recent studies have provided new insights into the structure of tetraspanins ([9–15], reviewed in [8]). In the CD9, CD81, and CD53 crystal structures, as well as in the Tspan15 cryo-EM structure (determined in association with ADAM10), the four transmembrane domains are arranged in two pairs of antiparallel helices (TM1/TM2 and TM3/TM4) that form a cone-shaped structure that converges near the cytoplasmic side of the membrane, creating a central intramembrane cavity (Figure 1). The large extracellular loop (LEL) bends toward the membrane and sits over the cavity formed by the transmembrane in a configuration referred to as the ‘closed’ structure [9–11,15]. Other studies have shown that tetraspanins can adopt an ‘open’ structure, in which the transmembrane domains do not form an intramembrane cavity and the LEL extends away from the membrane. This is the case of peripherin-2 (PRPH2) and rod outer segment membrane protein 1 (ROM1), two distant tetraspanins that form a heterodimer involved in photoreceptor morphogenesis [14]. CD81 also adopts an open conformation in the complex with CD19 (Figure 1 bottom, cryo-EM), showcasing the structural plasticity of this tetraspanin [13]. Additionally, the LEL of CD81 exhibits significant flexibility, as different crystal structures have revealed slightly varied arrangements, indicating some adaptability [16–18]. This structural versatility may enable CD81 to interact with a range of structural partners, including the E2 envelope protein of the hepatitis C virus for which CD81 serves as an obligatory receptor [19].

## Tetraspanins decorate and affect particular membrane structures

**Figure 2:**
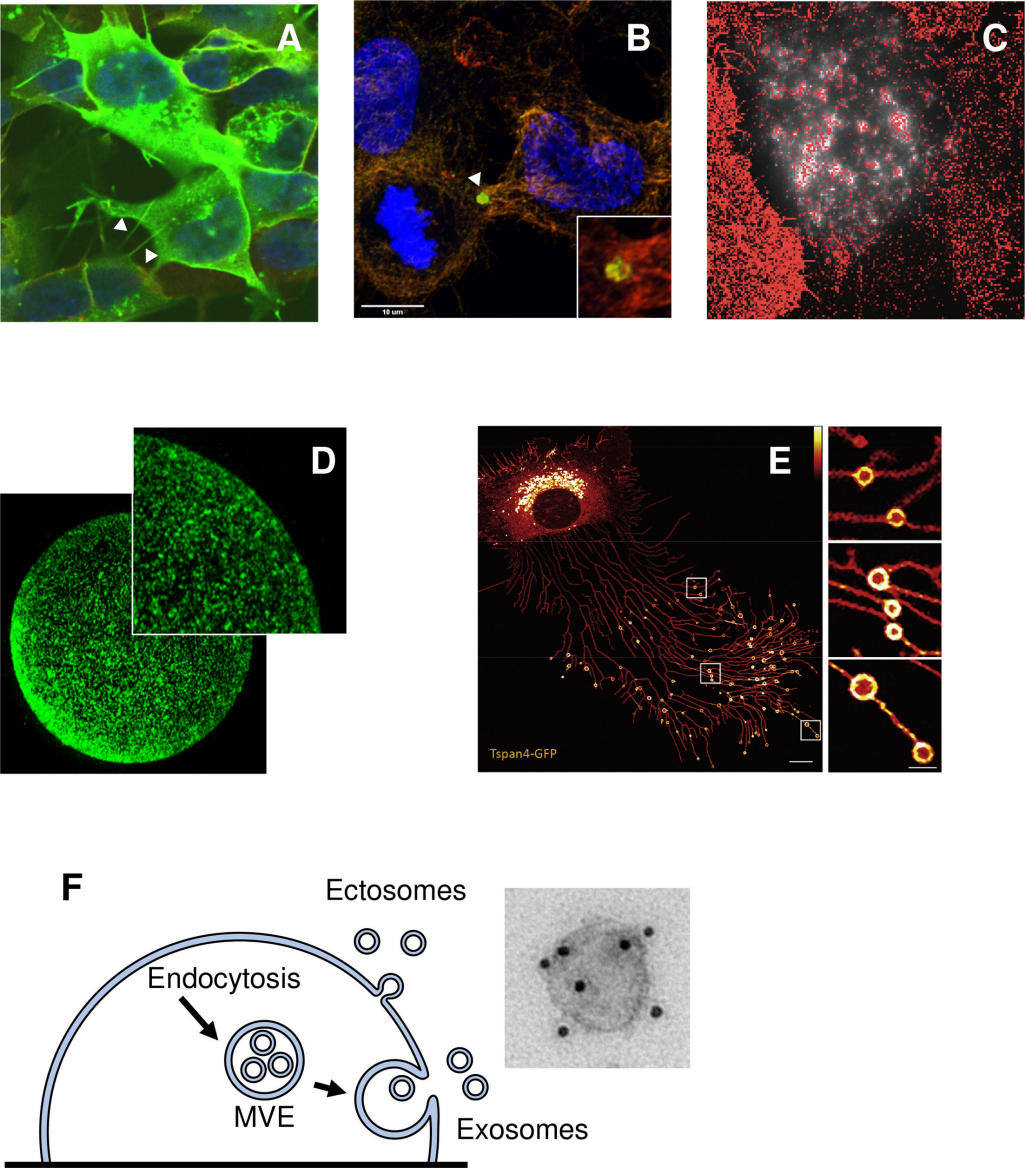
Examples of tetraspanin localization on remarkable membrane structures. (**A**): Localization of GFP-tagged CD9 on tunneling nanotubes in SH-SY5Y cells (image kindly provided by Dr. C Brou (see: [28]). (**B**): Localization of CD9 (red) and CD81 (green) on midbody remnants in HeLa cells, similarly to what has been described in [29]. (**C**): CD9 (red), detected by super-resolution microscopy concentrates in areas in which the HIV protein GAG (white) accumulates and induces membrane bending (image kindly provided by Dr. PE Milhiet; see: [30]). (**D**): CD9 expression in oocytes. The punctuated pattern reflects its preferential localization on microvilli as shown by [31]. (**E**): GFP-tagged Tspan4 localizes on retraction fibers and migrasomes. (image reproduced from [32]; https://doi.org/10.1016/j.cellin.2021.100003). (**F**): Small extracellular vesicles are released by either direct budding from the plasma membrane (ectosomes, enriched in CD9 and CD81) or following the fusion of multivesicular endosome resulting in the release of their intraluminal vesicles (exosomes, enriched in CD63). A labeling of CD63 on extracellular vesicle analyzed by electronic microscopy is also shown (image reproduced from [33], https://doi.org/10.3390/antib9030029).

While many tetraspanins, such as CD9, CD81, and CD151, are predominantly expressed at the cell membrane (see, e.g., among many others, [20–22]), others, like CD63 and Tspan6, show a prominent intracellular localization at steady state [23–25]. Many tetraspanins including CD9, CD81, and CD82 have been shown to be enriched in various membrane protrusions [26,27]. Among these protrusions, Tspan4 has gained recent attention because it is enriched (together with other tetraspanins) on retraction fibers, long tubular structures the cells leave behind them during migration, as well as migrasomes, micrometer-sized structures that form by local swelling of these fibers (Figure 2, [34]). Some tetraspanins are present on EVs released by cells into the extracellular space (Figure 2) [35], as well as on at least two enveloped viruses, influenza and HIV [36–41]. Among tetraspanins, CD9 and CD81 have been identified on both viruses, which is consistent with these two molecules being recruited to the site of HIV or influenza virus budding (Figure 2, [30,42]). A common feature between many of these cell structures is their high curvature, which aligns well with recent research using a biomimetic system of membrane tubules, which demonstrated that Tspan4 and CD9 sense membrane curvature and preferentially localize to regions with high positive curvature [43]. This behavior may be due to the cone-shaped structure of the tetraspanin intramembrane region [9–11].

Tetraspanins not only sense curvature but can also influence the shape of membrane structures. It has long been recognized that alterations in tetraspanin expression levels or the use of tetraspanin-targeting antibodies can lead to membrane remodeling (reviewed in [26,27]). A recent example is the demonstration that CD9 and CD81 are expressed on tubular nanotubes, long and thin membranous conduits between cells, and regulate their number or their ability to transfer material (Figure 2, [28]). A striking example is that of CD9, a tetraspanin essential for sperm–egg fusion [44], which localizes on egg microvilli (Figure 2) that become shorter and thicker in the absence of CD9 [31]. It is not known whether these modifications are the consequence of a direct effect of CD9 or secondary to cell signaling or cytoskeleton rearrangements. A potential direct effect of CD9 on membrane shape is suggested by the finding that in the CD9 crystals, the arrayed CD9 molecules in the lipidic environment triggered wavy layers in the crystalline lattice caused by the induction of curvature in the lipid membranes [10]. In addition, several tetraspanins have been shown to regulate migrasome formation and biomimetic *in vitro* models demonstrated that Tspan4 could generate or stabilize migrasome-like structures, particularly in the presence of cholesterol, suggesting a direct role for this tetraspanin in migrasome formation [34,45].

## Tetraspanins regulate the trafficking and subcellular localization of partner proteins

Many studies have investigated the role of tetraspanins, particularly in cancer, by identifying the proteins with which they associate, such as adhesion molecules, growth factor receptors, and ectoenzymes, and analyzing their influence on these proteins’ functions. Through these investigations, the most extensively studied tetraspanins, including CD9, CD81, CD82, CD53, CD151, and Tspan8, have been found to co-immunoprecipitate the same repertoire of integral membrane proteins and to associate with one another (reviewed in [3–5]). This observation led to the hypothesis that tetraspanins organize a dynamic network of interactions, referred to as the ‘tetraspanin web’ [46]. Tetraspanins have also been shown to partition into the light–density fractions of sucrose gradients, similarly to lipid-enriched microdomain (lipid raft) resident proteins, although under distinct experimental conditions. This finding led to the proposal that tetraspanins form tetraspanin-enriched microdomains, which could serve to cluster specific proteins and lipids, potentially playing a role in cell signaling [47,48]. This concept evolved into the broader idea that tetraspanins contribute to membrane compartmentalization [4,5]. It later became apparent that many of these interactions are likely indirect, partly secondary to tetraspanin–tetraspanin interactions [48–52]. Concurrently, the discovery of a limited number of well-characterized direct (primary) complexes [48–52] underscored the crucial and functionally relevant role of tetraspanins in regulating the trafficking and function of their direct interaction partners. This is particularly well illustrated by extensive studies on integrins, CD19, and ADAM10, which have been shown to associate with multiple tetraspanins but are distinctly regulated by the specific tetraspanins with which they directly interact.

Laminin-binding integrins such as α3β1, α6β1, and α6β4 interact directly with the tetraspanin CD151 [49,51]. CD151 silencing has been shown to reduce α3β1 integrin endocytosis without affecting the total or surface levels of the integrin, suggesting that CD151 influences integrin recycling in addition to internalization [53]. Moreover, CD151 regulates adhesion strengthening following the interaction of α6β1 integrin with laminin, a process essential for reinforcing the interaction between integrins and their ligands, allowing cells to withstand mechanical forces and avoid detachment [54]. This functional importance is highlighted by the phenotypes observed in patients with CD151 mutations or in CD151 knockout (KO) mice, which exhibit conditions similar to integrin deficiencies, such as skin blistering and kidney failure [55–57]. In the case of kidney failure, this defect is linked to the inability of podocytes, the filtering cells in the kidney’s filtration system, to resist the expansive forces generated by the pressure gradient within the kidney [57].

In contrast with CD151, CD81 is critical for the biosynthetic trafficking of its major partner protein in B lymphoid cells, the co-stimulatory molecule CD19, which is essential for optimal B-cell activation [58]. A patient with a CD81 mutation exhibited a severely impaired humoral immune response due to the absence of CD19 on the cell surface [59]. In mice, in which CD19 surface reduction is less pronounced, the humoral response was also affected and CD81 was shown to influence CD19 dynamics and organization [60,61]. As noted above, in a cryo-EM structure of CD81 associated with CD19, CD81 adopts an ‘open’ conformation, losing its intramembrane cavity as the LEL extends from the plasma membrane [13].

Another well-characterized example is the tetraspanin-dependent regulation of ADAM10. This metalloprotease is responsible for ectodomain shedding of various transmembrane proteins, which constitutes the normal secretion pathway for certain cytokine and growth factors, and is especially important for Notch signaling as it is the first step allowing the release of the Notch intracellular domain which acts as a transcriptional cofactor [62]. The efficient exit of ADAM10 from the ER requires the presence of one of a subset of six tetraspanins known as TspanC8, which are characterized by eight cysteines in their LEL [63–65]. TspanC8 tetraspanins not only facilitate ADAM10 egress from the ER but also regulate subsequent steps in its trafficking, membrane compartmentalization, and substrate selectivity [66–68]. Interestingly, TspanC8 tetraspanins are among the few tetraspanins with orthologs in invertebrates, which also regulate ADAM10 trafficking and Notch signaling in *Caenorhabditis elegans* and *Drosophila melanogaster* [64,69,70].

CD63, the best-characterized intracellular tetraspanin, traffics to the cell surface but is rapidly internalized due to a C-terminal GYxxΦ (GYEVM) motif that interacts with AP2 and AP3 adaptor proteins, targeting CD63 to late endosomes and lysosome-related organelles (LROs), including lysosomes and multivesicular endosomes (MVEs) [23]. Consistent with this, CD63 has been shown to address several interacting molecules to CD63-positive intracellular compartments, such as lysosomes. In its absence or when the GYxxΦ motif is mutated, these molecules localize at the plasma membrane. Examples of molecules the trafficking of which is regulated by CD63 include the H,K-ATPase β-subunit, colonic H^+^-K^+^ ATPase, Ca2^+^ sensor synaptotagmin VII, Pmel17, CXCR4, and MT1-MMP [71–76]. It remains unclear whether CD63 directly interacts with these proteins. Nevertheless, tetraspanins can also influence the trafficking of proteins with which they may not interact directly. For instance, Tspan7 has been shown to regulate AMPA receptor trafficking by interacting with the intracellular PDZ protein PICK1 through its PDZ-binding domain, competing with the AMPAR subunit GluA2 for PICK1 binding [77].

## Tetraspanins and extracellular vesicles

Cells secrete vesicles of various size delimited by a lipid bilayer that have been implicated in various physiological and pathological processes, including immunity, inflammation, neurodegeneration, and cancer. These EVs support intercellular communication through the exchange of proteins, lipids, and nucleic acids. EV signaling also occurs by contact with the plasma membrane of recipient cells [78,79]. The mechanisms of EV biogenesis vary between cell types and signaling status. Besides apoptotic bodies, two main subtypes of EVs—ectosomes and exosomes—have been categorized based on their subcellular origin. Ectosomes bud from the plasma membrane, and their diameter ranges from nanometer to micrometer scale. The formation of small ectosomes depends on endosomal sorting complexes required for transport (ESCRT) components governing membrane-budding and abscission and on the actin cytoskeleton. Exosomes are strictly nanosized vesicles that form upon budding of endosomal membranes, creating so-called intraluminal vesicles (ILVs) located inside MVEs. The fusion of MVEs with the plasma membrane releases the ILVs into the extracellular space as ‘exosomes’. Exosome biogenesis also depends on ESCRT and additional non-ESCRT components including ceramide, the PDZ domain-containing protein syntenin and syndecans. Yet one cannot exclude these components also contribute to the formation of small ectosomes [80].

It has long been known that EVs carry many tetraspanins. Following the initial demonstrations that CD9, CD37, CD53, CD63, and CD82 are present on EV released by immune cells [81,82], numerous proteomic analyses uncovered the presence of various tetraspanins on EVs (compiled in Vesiclepedia, http://microvesicles.org) [83]. Among them, CD9, CD81, and CD63, are widely used as markers of EVs. Consistent with its presence on ILVs [74,81], CD63 may preferentially decorate exosomes although it may also be present on ectosomes. In contrast, CD9 and CD81, which are mainly expressed at the plasma membrane, decorate ectosomes [21,84]. These two tetraspanins are also present on midbody remnants and migrasomes [29,34], which are released in the extracellular medium [85–87].

Several non-exclusive hypotheses have been explored regarding the roles of tetraspanins in EVs: (1) regulation of EV formation; (2) regulation of EV functional properties, including uptake and fusion; and (3) regulation of EV composition. However, current research has produced contradictory results, and a unified understanding of the function of tetraspanins in EVs remains elusive.

Some studies suggest that altering the expression of tetraspanins like CD9 or CD63 can affect the extent of EV release [88–91]. However, other studies found no such effects [22,84,92–95]. Moreover, the results are sometimes contradictory; for instance, CD63 was reported to positively influence EV release in some studies [88] but negatively in others [91]. These discrepancies suggest that tetraspanins are not essential components of the machinery responsible for EV formation. It is possible that the impact of these tetraspanins on EV release is dependent on the cellular context. In this regard, it was shown that the depletion of CD63 in melanocytes resulted in the formation of MVEs with lower number of ILVs [74]. This may be specific for this cell type and related to the process of melanogenesis, which occurs in LRO and depends on ILVs, since there was no modification of ILVs number in the absence of CD63 in another cell line [95]. The observed discrepancies might also stem from variations in isolation protocols or other technical differences, such as methods of quantification, purification, or characterization of EVs.

Given the influence of tetraspanins on specific cellular functions and their known interactions with various proteins, researchers have examined whether tetraspanins can regulate the sorting of particular proteins into EVs and the functions of EVs. For example, CD9 has been shown to interact with the ectopeptidase CD10/neprilysin and to enhance its release via EVs [96]. Additionally, overexpression of CD9 or CD82 was found to reduce Wnt/β-catenin signaling by increasing the release of β-catenin through EVs, an effect partly dependent on E-cadherin expression [97]. Similarly, Tspan8 expression in breast cancer cells was associated with increased levels of E-cadherin in EVs, probably as a consequence of the regulation of E-cadherin expression by Tspan8 [98]. In pancreatic cancer cells, Tspan8 transfection resulted in the targeting of the integrin α4 to EVs, enabling these EVs to bind to endothelial cells via VCAM1 and promoting angiogenesis [99].

Other studies have found that tetraspanins affect the functionality of EVs, which could indirectly suggest that they regulate EV composition or influence the function of specific components. For instance, Tspan8 transfection in breast cancer cells was shown to enhance EV attachment to recipient cells and stimulate their migration [100]. In another study, pre-incubation of cancer-associated fibroblast-derived EVs with a CD9 monoclonal antibody reduced their uptake by pancreatic cancer cells and their ability to stimulate cell migration [101]. However, it remains unclear whether this effect directly reflects the role of CD9 in this model. Elsewhere, transfection of CD9 in colon cancer cells was found to decrease EV uptake by recipient cells, potentially by regulating the interaction between EV-associated ADAM17 and the integrin α5β1 [102]. Yet, recent quantitative approaches suggest that the absence of CD9 or CD63 does not significantly alter the delivery of EV contents to recipient cells, indicating no major change in the uptake or internalization mechanisms [94].

Several studies have used proteomic approaches to analyze the composition of EVs in the absence of specific tetraspanins [22,90,93,95,103,104]. One study compared the composition of EVs secreted by lymphoblasts from wild type (WT) and CD81 KO mice [103]. Given that CD81 plays a crucial role in the immune system, particularly in the humoral response [105], the observed alterations in EV composition might stem from changes in lymphocyte subpopulations. This could explain the observed increase in CD4 and the decrease in B-cell molecules, such as CD20, immunoglobulins, or MHC class II antigens. Other studies examined EV protein composition in cell lines deficient in CD9, CD81, or CD63 [22,90,93,95,104]. While these studies reported several changes—both increases and decreases in protein levels—there were few commonalities, and some results were even contradictory. For instance, the absence of CD9 affected the secretion of proteasome subunits differently across studies, with PSMB7 showing a decrease in one study, an increase in another, and no change in a third [22,90,93]. These changes, along with changes in metabolic enzymes or ribosomal/RNA-binding proteins, are unexpected since these proteins are not typically found in tetraspanin-decorated EVs [106]. This suggests that some observed changes might be indirect effects of tetraspanin absence, or perhaps directly related to the experimental or analytical methods used, reflecting variations in the methods for isolation, quantification or characterization of EVs used in the different studies. Overall, these findings suggest that CD9, CD81, and CD63 do not play a general role in sorting protein cargos into EVs. However, it is possible that certain effects of tetraspanin deletion went undetected due to potential functional redundancies among tetraspanins. In this regard, one study [22] found that two immunoglobulin domain proteins, CD9P-1/EWI-F (encoded by the PTGFRN gene) and EWI-2 (encoded by the IGSF8 gene), which directly associate with both CD9 and CD81 [3–5], were decreased in EVs lacking both tetraspanins, but not only one of them. While the reduction in EWI-2 was primarily due to a lower cellular level of the protein, the decrease in CD9P-1 was not, indicating that CD9 and CD81 contribute to its sorting into EVs. In contrast, β-catenin levels in EVs were unaffected by the absence of CD63, CD9, CD81, or both CD9 and CD81. This suggests that the CD9-dependent regulation of β-catenin targeting to EVs, previously reported [97], is not a general phenomenon.

Tspan6 has gained significant attention recently for its role in EV formation. Similar to CD63 and CD81 [107,108], Tspan6 interacts directly with the scaffolding PDZ protein syntenin [24,25] that plays an important role in the formation of EVs [92,109]. In HEK 293 cells, Tspan6 primarily resides after transfection in LAMP1-positive organelles, akin to CD63, making it a resident of LROs. Overexpression of Tspan6 in these cells was shown to alter endosomal structures and enhance EV release, an effect not observed in syntenin-deficient cells [24]. However, contrasting results were observed in breast cancer MCF7 cells, where Tspan6 limited EV release by promoting syndecan-4-dependent syntenin lysosomal degradation [25]. Further complicating the picture, transfection of Tspan6 in another breast cancer cell line did not alter EV release [110], and organoids derived from APC mutant Tspan6 KO mice produced similar amounts of EVs as those expressing Tspan6 [111]. Importantly, in this latter study, Tspan6 was found to interact with TGFα via syntenin, and its absence led to increased TGFα release in EVs, making organoid growth independent of exogenous EGF and enhancing tumor formation in APC mutant animals.

Tetraspanins have long been recognized for their physical and functional interactions with lipids, such as cholesterol and gangliosides like GM3 [112–117]. The crystal structure of CD81, generated in the presence of cholesterol, revealed the presence of this lipid in the central cavity formed by the transmembrane domains [9]. Several studies have explored how tetraspanin absence affects the lipid composition of EVs [90,95,110]. Following the discovery that Tspan6 enhances the chemoattractive potential of breast cancer cells for B lymphocytes in an EV-dependent manner, it was shown that this effect relied on the function of liver X receptors, which are nuclear receptors for oxysterols, in B cells. Tspan6 expression was associated with increased levels of oxysterol species (25-HC and 27-HC) in EVs as well as in crude cellular membranes, without any change in the total cell oxysterol levels suggesting that it participates to the transport of oxysterols [110]. Importantly, a mutation in Tspan6 predicted to disrupt oxysterol binding abolished its ability to elevate oxysterol levels in EVs and stimulate B-cell migration.

Molecular modeling also suggests that CD63 could accommodate cholesterol within an intramembrane cavity [95]. Although the absence of CD63 did not affect the uptake of exogenous cholesterol from LDL, it altered the intracellular trafficking of endogenous cholesterol. Specifically, CD63 deficiency resulted in reduced labeling of ILVs in MVEs by the D4 fragment of the cholesterol-binding toxin perfringolysin O, alongside increased Golgi apparatus labeling. EVs from CD63-KO cells also exhibited reduced D4 labeling, which was fully reversed by reintroducing CD63 but not by expressing a mutant CD63 predicted not to accommodate cholesterol in its intramembrane cavity. As not all approaches indicated a decreased cholesterol level in EVs released by CD63 KO cells, it is possible that D4 detects discrete cholesterol pools modified by the absence of CD63. Importantly, EVs from CD63 KO cells loaded with a fluorescent analogue of cholesterol delivered less of this fluorescent probe to recipient cells than EVs from WT cells.

## Conclusion

Tetraspanins have long been associated with a wide range of cellular functions and molecular interactions. Their ability to directly interact with partner proteins has led to the emerging concept of tetraspanins as regulators of cell membrane compartmentalization. Recent structural studies of full-length tetraspanins provide insight into their localization in and their impact on curved and tubular cellular structures. Small EVs represent a typical example of curved structures enriched in several tetraspanins. Early hypotheses suggesting that discrete tetraspanins play a role in EV formation or protein composition have been challenged by conflicting experimental data. However, recent studies indicate a possible role for tetraspanins in the trafficking and/or organization of sterols within EVs. These findings open new avenues for research into how tetraspanins influence the lipid composition of EVs.

**Perspectives**

- Many cells release extracellular vesicles (EVs) in their environment, which often bear tetraspanins such as CD9, CD81, and CD63. These three tetraspanins are commonly used as markers of EVs although some EVs do not carry any of them.
- The ability of tetraspanins to regulate the trafficking of associated partner proteins and to have an impact on curved or tubular structures, possibly due to their conical structure, may suggest a role in the formation or protein composition of EVs. However, these hypotheses have been confounded by contradictory results.
- Future studies could revisit these hypotheses by analyzing discrete subtypes of EVs and could seek to confirm the impact of tetraspanins on the composition or organization of lipids in EVs.

## Abbreviations

ESCRT: endosomal sorting complexes required for transport
EVs: extracellular vesicles
ILVs: intraluminal vesicles
LEL: large extracellular loop
LRO: lysosome-related organelle
MVEs: multivesicular endosomes/bodies
PRPH2: peripherin-2
ROM1: rod outer segment membrane protein 1
